# Human Neutrophil Peptide-1 (HNP-1): A New Anti-Leishmanial Drug Candidate

**DOI:** 10.1371/journal.pntd.0002491

**Published:** 2013-10-17

**Authors:** Sara Dabirian, Yasaman Taslimi, Farnaz Zahedifard, Elham Gholami, Fatemeh Doustdari, Mahdieh Motamedirad, Shohreh Khatami, Kayhan Azadmanesh, Susanne Nylen, Sima Rafati

**Affiliations:** 1 Molecular Immunology and Vaccine Research Laboratory, Pasteur Institute of Iran, Tehran, Iran; 2 Virology Department, Pasteur Institute of Iran, Tehran, Iran; 3 Biochemistry Department, Pasteur Institute of Iran, Tehran, Iran; 4 Department of Microbiology Tumor and Cell Biology (MTC), Karolinska Institutet, Stockholm, Sweden; Queensland Institute of Medical Research, Australia

## Abstract

The toxicity of available drugs for treatment of leishmaniasis, coupled with emerging drug resistance, make it urgent to find new therapies. Antimicrobial peptides (AMPs) have a strong broad-spectrum antimicrobial activity with distinctive modes of action and are considered as promising therapeutic agents. The defensins, members of the large family of AMPs, are immunomodulatory molecules and important components of innate immune system. Human neutrophil peptide-1 (HNP-1), which is produced by neutrophils, is one of the most potent defensins. In this study, we described anti-parasitic activity of recombinant HNP-1 (rHNP-1) against *Leishmania major* promastigotes and amastigotes. Furthermore, we evaluated the immunomodulatory effect of rHNP-1 on parasite-infected neutrophils and how neutrophil apoptosis was affected. Our result showed that neutrophils isolated from healthy individuals were significantly delayed in the onset of apoptosis following rHNP-1 treatment. Moreover, there was a noteworthy increase in dying cells in rHNP-1- and/or CpG–treated neutrophils in comparison with untreated cells. There is a considerable increase in TNF-α production from rHNP-1-treated neutrophils and decreased level of TGF-β concentration, a response that should potentiate the immune system against parasite invasion. In addition, by using real-time polymerase chain reaction (real-time PCR), we showed that *in vitro* infectivity of *Leishmania* into neutrophils is significantly reduced following rHNP-1 treatment compared to untreated cells.

## Author Summary

In Iran, cutaneous leishmaniasis (CL) is a widespread and highly endemic disease in young individuals. To date, treatment strategy is based on chemotherapy accompanied with high incidence of toxicity and drug resistance. Distinctive mode of action of defensins (members of antimicrobial peptides) with low susceptibility to resistance and low toxicity to mammalian cells makes them suitable candidates for anti-leishmanial agents. The most active human defensin is human neutrophil peptide-1 (HNP-1) produced by neutrophils; the first effector cells during *Leishmania* infection. In this work, we used recombinant HNP-1 (rHNP-1) against both the promastigote and amastigote forms of *Leishmania (L.) major*. Furthermore, immunomodulatory effect of rHNP-1 on *Leishmania*-infected neutrophils was investigated. Our result showed that rHNP-1 has anti-parasitic effect against *L. major* promastigotes and amastigotes and also reduces infectivity rate of *Leishmania*-infected neutrophils. Moreover, assessment of cytokine production from *Leishmania*-infected neutrophils reveals an increase in TNF-α and a decrease in TGF-β production after rHNP-1 treatment; a cytokine pattern anticipated to facilitate control of parasites. The immunomodulatory effect of rHNP-1 on cytokine production from parasite-infected neutrophils besides its direct effect on free parasites is considered as promising step towards developing new anti-leishmanial agents.

## Introduction

AMPs are small, cationic proteins which are found in a wide variety of organisms and function as key components of the innate immune system [1], [2]. They exhibit broad-spectrum anti-bacterial, anti-viral, anti-fungal and anti-parasitic activities and have low cytotoxicity to mammalian cells at concentrations required to kill microorganisms [3], [4], [5], [6], [7], [8], [9]. Positive charge together with amphipathicity enable AMPs to interact with negatively-charged microbial surface membranes leading to permeation, disruption and ultimately cell death [2]; a mechanism considerably different from currently available anti-leishmanial drugs. These qualities combined with low susceptibility to resistance makes AMPs good candidates as anti-leishmanial agents [3].

Defensins belong to a large family of AMPs including cysteine-rich peptides with three or four intra-molecular disulfide bonds. They are classified as α-, β- and θ-defensins [10] where the first two are the most common human antimicrobial peptides. The α-defensins found in neutrophils (polymorphonuclear cells, PMNs) are called human neutrophil peptides (HNP)-1, -2, -3 and -4 [11]. Neutrophils constitutively express α-defensins with an increase in production level during infections [12], [13], [14]. In addition to being potent antimicrobial agents, HNPs may act as immunomodulatory molecules since they induce cytokine production and immune cell activation [15].

In cutaneous leishmaniasis (CL), neutrophils are like a double-edged sword. On the one hand, they are believed to be the first recruited effector cells right after infection [16]. Their increased accumulation (as a result of defensins and/or other stimulators) may evoke an immune response set to control *Leishmania* infection. On the other hand, they may be exploited by the parasites and the presence of neutrophils has been found to facilitate infection experimentally [17]. Thus, understanding the neutrophil-parasite interaction may be an important step towards understanding the underlying mechanisms controlling the parasite. We addressed this interaction by characterization of changes in the production of different cytokines, including tumor necrosis factor-α (TNF-α), Interleukin-8 (IL-8) and transforming growth factor-β (TGF-β). TNF-α is a pro-inflammatory cytokine that causes differentiation and activation of dendritic cells (DC) and macrophages [18] and contributes to intracellular parasite elimination by neutrophils [19]. IL-8 is an important neutrophil chemotactic attractants [18] that induces degranulation of toxic granules allowing neutrophils to kill invading microorganisms [20]. TGF-β, on the other hand, is an immunosuppressive cytokine, beneficial for parasite persistence within neutrophils and a suppression of T-helper 1 type (Th1) response; the protective response against CL.

Being abundant in azurophilic granules of human neutrophils; the primary effector cells against cutaneous infection with *Leishmania* [21], [22] and being considered as the most active human α-defensins [23], we decided to investigate the *in vitro* activity of recombinant HNP-1 (rHNP-1) against *L. major*. Using a prokaryotic expression system and an *in vitro* folding procedure, we succeeded to produce substantial amount of active peptide. Beside *in vitro* evaluation of rHNP-1 effect on stationary-phase promastigote and amastigote forms of *L. major*, we assessed the effect of rHNP-1 in combination with CpG motif on human neutrophil's lifespan and cytokine production pattern. We have previously shown that CpG motifs of class A is superior to class B, in induction of TNF-α production [24]. Therefore, in this study, we harnessed class A CpG motif besides rHNP-1 for investigating their immunomodulatory effects. *In vitro* cytokine assay showed a considerable increase in TNF-α production from rHNP-1-treated neutrophils and decrease of TGF-β concentration in rHNP-1- and CpG-treated cell cultures. Furthermore, a decreased infectivity following rHNP-1 treatment of human neutrophils was another important effect on the parasite. Moreover, we found that rHNP-1 changes the lifespan of neutrophils in a way anticipated to favor parasite clearance. Our results favor further investigation on AMPs as a new class of anti-leishmanial agents.

## Materials and Methods

### CpG motif

Class A CpG motif (ggT GCA TCG ATG CAG ggg gg) used in all experiment was synthesized by TIB MOLBIOL Syntheselabor GmbH (Germany). Bases in capital letters were modified with phosphorodiester and those in lower-case letters with phosphorothioate respectively.

### Parasite culture

*L. major* (MHRO/IR/75/ER) was cultivated *in vitro* in M199 medium (Sigma, Germany) supplemented with 5% heat-inactivated fetal calf serum (FCS) (GIBCO BRL, Germany), 40 mM HEPES, 0.1 mM adenosine, 0.5 µg/ml hemin and 50 µg/ml gentamicin (all from Sigma, Germany) and was maintained at 26°C. Stationary-phase promastigotes (6-day-old) were harvested and washed in phosphate buffered saline (PBS) prior to use.

### Ethics statement

All blood samples from healthy volunteers were collected following written informed consent, according to institutionally approved procedures (Pasteur Institute of Iran ethical committee, approved on 2nd of October 2009)

### Neutrophil isolation

Whole blood (20 ml) was obtained from healthy volunteers (11 women and 9 men, age 25–60 years). Polymorphprep (Axis-Shield Poc Ac, Norway) was used to isolate polymorphonuclear granulocytes from whole blood, following instructions from manufacturer. The isolated granulocyte population contained 98% neutrophils and 2% eosinophils and there are no lymphocytes or monocytes as determined microscopically after Kimura staining. This staining method enables the discrimination between neutrophils and eosinophils [25]. Isolated neutrophils had 98% viability as assessed by trypan blue dye exclusion. Isolated cells were cultured in RPMI 1640 medium (Sigma, Germany) supplemented with heat-inactivated FCS (10%), HEPES (1%), L-glutamine (1%, Sigma, Germany) and 100 µg/ml gentamicin.

### Isolation of total PMN RNA and preparation of cDNA

RNeasy mini kit (Qiagen, Germany) was used for isolation of total RNA from purified PMNs based on manufacturer's protocol. The quality of the RNA was assessed by gel electrophoresis on a 1% agarose gel. RNA was then reverse-transcribed into cDNA using oligo-dT primers based on instructions from Omniscript Reverse Transcription kit (Qiagen, Germany).

### HNP-1 cloning and DNA sequencing

According to HNP-1 nucleotide sequence data registered in GenBank (NM_004084.3) and by means of primer express program, specific complementary set of forward (5′-TATGGATCCGTCGACATGGCCTGCTAT-3′) and reverse (5′-AATGAGCTCGGTACCGCAGCAGAATGC-3′) oligomers flanked with *BamH*I and *Sac*I restriction enzyme sites were designed. 50 ng of cDNA and specific primers (10 pmol per reaction) plus 0.5 µl Taq DNA polymerase (Roche, Germany) were used in the PCR reaction mixture (30 µl). Amplification steps included 95°C for 5 min followed by 40 cycles of 95°C for 15 sec, 63°C for 20 sec, 72°C for 40 sec and 72°C for 10 min as the final step. PCR products were analysed by 2% agarose gel electrophoresis with ethidium bromide staining. Product of the PCR assembly containing HNP-1 coding sequence flanked with *BamH*I and *Sac*I was purified by a gel extraction kit (QIAquick Gel Extraction kit, Qiagen, Germany) and ligated into the cloning vector pGEM-2 (Promega, USA). The recombinant vectors were transformed into competent DH5α cells. In order to identify colonies including pGEM-HNP-1, PCR and enzymatic digestion were carried out. After isolation and purification, the obtained clone was confirmed by DNA sequencing analysis.

### HNP-1 expression

After verifying DNA sequence, HNP-1 was ligated into the expression vector pQE-30 (Qiagen, Germany). The resulting recombinant vector (pQE-HNP-1) was transformed into the *E. coli* M15 cells. After confirmation by PCR and enzymatic digestion, a clone was chosen (pQE-HNP-1) and used in overnight cultivation at 37°C in Luria-Bertani (LB) broth medium supplemented with ampicillin (0.1 mg/ml, Jaber Ibn Hayan, Iran) and kanamycin (0.025 mg/ml, Sigma, Germany). The overnight culture was then used to inoculate the fresh LB medium (supplemented with antibiotics) at 37°C. Peptide expression was induced by adding 1 mM isopropyl-β-D-thiogalactoside (IPTG, Roche, Germany), at optical density of 0.75 and the culture was grown for another 4 hours. 17.5% standard sodium dodecyl sulphate polyacrylamide gel electrophoresis (SDS-PAGE) and Western blot analysis using anti-His antibodies (Qiagen, Germany) was used to check peptide expression.

### HNP-1 purification on nickel-nitrilotriacetic acid (Ni-NTA) column by Fast Protein Liquid Chromatography (FPLC)

In order to determine HNP-1 solubility, after 4-hour post IPTG induction, the bacterial pellet was suspended in a buffer containing NaH_2_PO_4_ (50 mM), NaCl (300 mM) and imidazole (10 mM) (all from Merck, Germany), and then lysozyme (1 mg/ml, Roche, Germany) was added to the suspension. After 30 min incubation on ice (to destroy the cell wall), the sample was sonicated and centrifuged. Supernatant (soluble extract) and pellet (insoluble extract) were separately evaluated by SDS-PAGE to determine the solubility of recombinant peptide.

The 4-hour bacterial pellet distinguished to harbor the highest amount of expressed peptide was dissolved in lysis buffer containing Tris-base (Sigma, Germany) solution (50 mM) and NaCl solution (100 mM), incubated on ice for an hour and sonicated by ultrasonic processor (Cole-Parmer, CPX 750, USA). Then, the suspension was centrifuged at 500 g for 20 min followed by centrifuging supernatant at 12000 g for 30 min. The resulted pellet was resuspended in a denaturating buffer containing 8M urea (Sigma, Germany) and 20 mM imidazole and placed on rocker for 1 hour. Denatured peptide was further purified on Ni-NTA resins through His-tag residues (encoded by pQE-HNP-1 construct). After centrifuging at 12000 g for 30 min, the supernatant was loaded on Ni-NTA column, HiTrap chelating HP (Amersham Biosciences, Sweden). In this FPLC system, two buffers containing 0 mM and 500 mM imidazole along with 8M urea were used for stepwise extraction. 30 mM imidazole buffer was first applied to wash the column. Then, the concentration of imidazole was gradually increased to 350 mM. At this concentration, the HNP-1 dissociated from the column and appeared in the effluent and was collected for further use. In all steps, pH was adjusted to 8. The purified peptide was characterized by MALDI-TOF/TOF mass spectrometry (4700 Proteomics Analyzers, Applied Biosystems, UK).

### Folding of HNP-1

For peptide folding, we followed the method described by Rehder and Borges [26]. Since the peptide was purified in 8M urea, urea was removed to obtain proper folding. Briefly, purified, unfolded HNP-1 was loaded into an Amicon Ultra-15 (3 kDa MWCO) centrifugal concentration unit (Millipore, France) containing 4 ml of 0.1M acetic acid. The sample was centrifuged in swing-bucket rotor for 40 min at 3000 *g*. The retentate was then rediluted with 4 ml of 0.1M acetic acid. Repeating the cycle a total of seven times, resulted in urea dilution more than 10^6^-fold. Then a solution of 0.1M acetic acid containing 50 µM CuSO_4_ was prepared and adjusted to pH 8 with a saturated solution of Tris-base. An aliquot of the retentate was diluted to 75 µg/ml with the prepared solution and left to spontaneously refold at room temperature in the presence of air for 16 hours.

### Anti-bacterial activity of rHNP-1

In order to confirm effective folding, its anti-bacterial activity was investigated in comparison with commercial human alpha-Defensin-1 (commercial HNP-1, Peptide Institute, Japan) against *E. coli* (ATCC 25922) as a susceptible target. The anti-bacterial activity was determined using a standard protocol against *E. coli* cells described by Pazgier and Lubkowski [27].

### Anti-promastigote assay

In this assay, the ability of rHNP-1 (with different concentrations ranging from 0 to 60 µg/ml) to kill stationary-phase promastigotes of *L. major* was evaluated. Furthermore for comparing the activity of rHNP-1 with its commercial form, the effect of two concentrations (10 and 40 µg/ml) of commercial HNP-1 was evaluated on *L. major*. 50 µl stationary-phase promastigotes (10^7^/ml) were resuspended in sterile PBS and incubated in duplicate with rHNP-1 and its commercial form for 16 hours in a 96-well plate to evaluate the parasiticidal activity of the peptides. Untreated control parasites received mock treatment with sterile Milli-Q water (Milli-Q System, Millipore, France). 25-µl aliquot of each sample was diluted in 275 µl PBS and stained with 3 µl propidium iodide (PI, Biovision, USA) based on manufacturer's protocol, followed by flow cytometric (FASC) analysis (Partec CyFlow, Germany) to determine the percentage of PI-stained promastigotes in the population. 50000 events were analyzed for each sample using Flomax software (Partec, Germany).

### Amastigote detection assay

LM1 cell line (kind gift of M. Olivier, McGill University, Department of Microbiology and Immunology), immortalized bone marrow-derived macrophages from motheaten mice, was seeded in a 12-well plate at a density of 2×10^5^/ml in serum free DMEM medium (Sigma, Germany), and incubated for 16 hours at 37°C in a humidified atmosphere containing 5% CO_2_. Non-adherent cells were removed and 1 ml of fresh DMEM medium supplemented with 5% FCS was added to each well. Cells were then infected by stationary-phase promastigote of *L. major* at 5∶1 parasite to macrophage ratio and incubated for further 16 hours. After that, each well was washed three times by PBS to remove free parasites. Then, cells were treated by 20 µg/ml of rHNP-1 or unfolded HNP-1 followed by a further incubation for 16 hours in the same condition. Culture as control (without rHNP-1 treatment) was included. Cells were collected and subject to genomic DNA extraction using DNA Extraction Kit (Qiagen, Germany) according to manufacturer instructions. The obtained genomic DNA was quantified by a spectrophotometer (Nanodrop, ND-1000, USA) and 150 ng of each sample was subject to real-time PCR using a 7500 Real-Time PCR System (Applied Biosystems, USA) in order to quantify DNA polymerase II (DNApol), the target gene of the parasites, and TATA-binding protein (TBP), one of the house keeping genes of macrophages by use of specific forward and reverse primers. The primers used for DNApol amplification were sense 5′-CGCCTTGTTGTGGACTCCTACT-3′ and antisense 5′-TGTTGCTGCCCTTTGTAATCC-3′. Those used for TBP amplification were sense 5′-AGTTGTCATACCGTGCTGCTA-3′ and antisense 5′-TTCTCCCTCAAACCAACTTGTCA-3′. A PCR volume of 25 µl included 2.5 µl DNA template, 9 µl QuantiFast SYBR Green PCR master mix (Qiagen, Germany), 1 µl of sense and antisense primer (10 pmol per reaction) and 11.5 µl DNase free water. The PCR program was initiated at 95°C for 10 min, followed by 40 thermal cycles of 15 sec at 95°C, 30 sec at 60°C and 40 sec at 72°C. Alongside each real-time PCR assay, a series of standards was prepared by performing 10-fold serial dilutions of recombinant pDrive cloning vector (Qiagen, Germany) encoding DNApol or TBP and covering a range of 2500 to 2.5×10^7^ copies per PCR reaction. This series of standards served as a calibrator and the amounts of DNApol and TBP in each sample was calculated from the generated standard curve at the end of the assay (the regression line for the standards had always an R^2^>0.99). As a negative control, reactions without DNA template were also included. For each sample, PCR was performed in duplicate, and each experiment was performed three times. DNApol quantity was normalized to TBP quantity of each sample.

### Cell viability assay

In order to determine the cytotoxic effect of rHNP-1 on neutrophils and LM-1 cell line, we performed methylthiazol tetrazolium (MTT) assay 24 hours after incubation of cells with rHNP-1. Briefly, 1.5×10^6^ neutrophils per well were seeded in a 96-well plate in complete RPMI 1640 medium and treated with different concentrations of rHNP-1 (in the range of 0 act as control up to 60 µg/ml) followed by incubation for 24 hours at 37°C in a humidified atmosphere containing 5% CO_2_. In the case of LM-1 cell line, cells were seeded in a 96-well plate at the density of 5×10^4^ per well in DMEM medium (without FCS), and incubated for 16 hours at 37°C in a humidified atmosphere containing 5% CO_2_. Non-adherent cells were removed and then cells were treated with different concentrations of rHNP-1 (as mentioned for neutrophils) in complete DMEM medium followed by incubation for 24 hours in the same condition. Cells were then washed twice with PBS, and 180 µl of fresh medium (without FCS) and 20 µl of MTT (Sigma, Germany) solution (5 mg/ml in PBS) were added to each well, followed by incubation for an additional 4 hours. The supernatants were removed, and 150 µl of DMSO (Sigma, Germany) was added to each well. After complete dissolution of formazan crystals, the optical density (OD) of the solution was measured by an absorbance microplate reader (BioTek, USA) at 570 nm using a reference wavelength of 630 nm. The percentage of cell viability was determined based on following equation: (OD_treated group_/OD_control group_)×100. Experiments were performed three times in triplicate.

### Assessment of neutrophil apoptosis

Neutrophils isolated from healthy volunteers were seeded in a 12-well plate at a density of 2×10^6^ cells per well in RPMI 1640 medium supplemented with 10% FCS. Half of the wells were infected by stationary-phase promastigotes of *L. major* at a parasite-to-PMN ratio of 5∶1 and the other half remained uninfected followed by 4-hour incubation at 37°C in a humidified atmosphere containing 5% CO_2_. Cells were then washed three times with PBS and 1 ml fresh medium was added to each well. All cells (uninfected and infected groups) were treated by 20 µg/ml of rHNP-1 (in the presence or absence of 20 µg/ml of class A CpG motif) followed by a further incubation for 18 hours in the same condition. Apoptosis was assessed by flow cytometry using fluorescein isothiocyanate (FITC)–annexin V and PI (Biovision, USA), according to the manufacturer's instructions. After 10-min incubation in dark, cells were subject to flow cytometric analysis on a Partec CyFlow cytometer. FSC and SSC were used to identify the PMN population. 15000 events were counted per sample and the percentages of apoptotic, dead or viable cells were determined. Apoptotic neutrophils were defined as annexin V-positive but PI-negative cells, dead neutrophils were defined as annexin V-positive and PI-positive cells and viable neutrophils were defined as annexin V-negative and PI-negative cells. This assay was performed on isolated PMNs from 10 healthy individuals (in ten separate experiments) and in duplicate.

### Cytokine assay and genomic DNA preparation

The effect of different concentrations of granulocyte-macrophage colony stimulating factor (GM-CSF) on cytokine production by neutrophils was investigated in our previous study [24] and a concentration of 50 ng/ml was considered as optimal. To determine cytokine production from rHNP-1- and/or CpG-treated cells, isolated neutrophils (2×10^6^ cells per well) from 20 healthy volunteers were seeded in 12-well plates in RPMI 1640 medium supplemented with 10% FCS and incubated in the presence of 50 ng/ml of recombinant human GM-CSF (RELIA*Tech* GmbH, Germany) at 37°C in a humidified atmosphere containing 5% CO_2_ for 90 minutes. The supernatants were then removed and half of the wells were infected by stationary-phase *L. major* promastigotes at a parasite-to-PMN ratio of 5∶1 and the other half remained uninfected followed by additional 4-hour incubation. After washing the cells with PBS three times, all cells (uninfected and infected groups) were treated by 20 µg/ml of rHNP-1 (in the presence or absence of 20 µg/ml of class A CpG motif) followed by a further incubation at 37°C for 18 hours (some wells containing infected neutrophils were added unfolded HNP-1, as control). Culture supernatants were then collected and stored at −80°C until use for quantification of TNF-α, IL-8 and TGF-β by enzyme-linked immunosorbent assay (ELISA). All cytokines were quantified using DuoSet ELISA kits (R&D Systems, Germany) according to the manufacturer's instructions.

After collecting supernatants, untreated, infected neutrophils and those treated by rHNP-1 and unfolded HNP-1 were collected and were subject to genomic DNA extraction using DNA Extraction Kit (Qiagen, Germany) according to manufacturer's instruction. For further study, neutrophil samples related to 10 individuals (out of 20) were randomly chosen and subject to genomic DNA extraction.

### Infectivity rate of *Leishmania* in PMNs after rHNP-1 treatment

Real-time PCR assay was run to test the potential of rHNP-1 on reducing infectivity rate of *Leishmania*-infected PMNs *in vitro*. Extracted genomic DNA from each sample was quantified (Nanodrop, ND-1000, USA) and 100 ng of each sample was subject to real-time PCR in order to quantify DNApol, the target gene of the parasite, by means of specific forward and reverse primers (previously mentioned in amastigote detection assay section). PCR condition was the same as mentioned. For each sample, PCR was performed in duplicate, and each experiment was performed twice. The percentage of infectivity rate reduction was determined based on following equation: [1−(DNApol quantity_treated group_/DNApol quantity_control group_)]×100.

### Statistical analysis

GraphPad Prism (version 5, GraphPad Software, USA) was used for all statistical analyses. The results are shown as mean ± standard deviation (SD). A *p*-value of less than 0.05 was considered as statistically significant difference.

## Results

### Isolation of total RNA from obtained PMNs and cloning of HNP-1

As shown in Figure S1A, isolated RNA exhibited a desirable quality. Purity of total RNA was evaluated with Agilent 2100 Bioanalyzer according to our previous study [24]. RNA was then reverse-transcribed into cDNA. 2% agarose gel electrophoresis exhibited an expected single band of 123 bp (Figure S1B) in accordance with its calculated length. DNA sequencing of pGEM-HNP-1 clone showed that the gene sequence of interest was in-frame correctly.

### Expression and purification of HNP-1 on Ni-NTA column by FPLC

**Figure 1:**
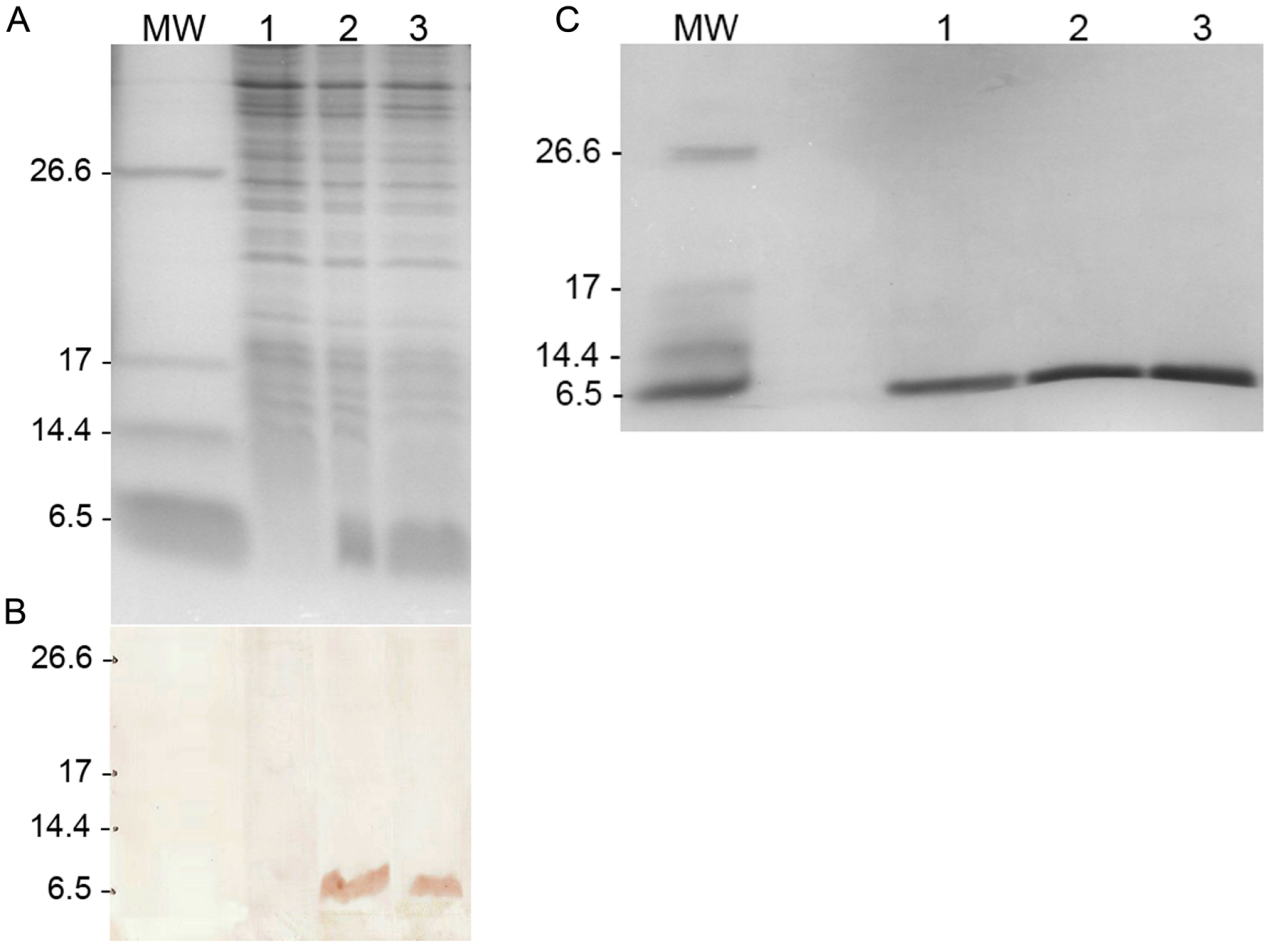
Expression and purification of rHNP-1. A) Expression of rHNP-1 in pQE-30. SDS-PAGE analysis of the target peptide showed that HNP-1 was successfully expressed. B) Western blotting analysis of the expressed peptide. MW: 100-bp molecular weight marker. lane 1: before IPTG induction; lane 2 and 3: 4 hours after induction. C) HNP-1 purification on Ni-NTA column by FPLC. The identity and purity of each FPLC fraction were evaluated on 17.5% SDS-PAGE. As illustrated in this figure, the peptide elutes (lanes 1–3) had acceptable purity for further use. MW: 100-bp molecular weight marker; lane 1, 2 and 3: peptide elutes.

Expression of peptide was evaluated on 17.5% SDS-PAGE (Figure 1A) and confirmed by Western blot analysis using anti-His antibodies as shown in Figure 1B. The molecular weight of obtained peptide is about 6.5 kDa. 4 hours post IPTG induction by comparing the soluble and insoluble extracts on SDS-PAGE, we found the majority of peptide to be presented in the insoluble phase. For further purification of HNP-1 in insoluble extract, FPLC was performed as described in M&M. The obtained isolated fractions were evaluated on 17.5% SDS-PAGE (Figure 1C) and deemed to be of sufficient purity for further use. Moreover, the mass spectrometry analysis and Triptic digestion showed 63% sequence coverage with theoretic sequence (score = 206). The purified HNP-1 was folded and kept at −70°C for further use.

### Evaluation of anti-bacterial activity of rHNP-1

To confirm effective folding of the rHNP-1, anti-bacterial activity of rHNP-1 was investigated against *E. coli* in comparison with commercial HNP-1 using standard assay of Pazgier and Lubkowski [27]. The results of these experiments are shown in Figure S2. As the bacteria inhibition curve illustrates in this Figure, the growth of *E. coli* was dramatically suppressed with the increasing concentrations of rHNP-1 and its commercial form. The potency of commercial HNP-1 against *E. coli* at highest (16 µg/ml) and lowest concentrations were 7-fold and 1.1-fold more than recombinant form respectively (as illustrated in Figure S2, *p*<0.05).

### HNP-1 cause necrosis and has anti-promastigote activity

**Figure 2:**
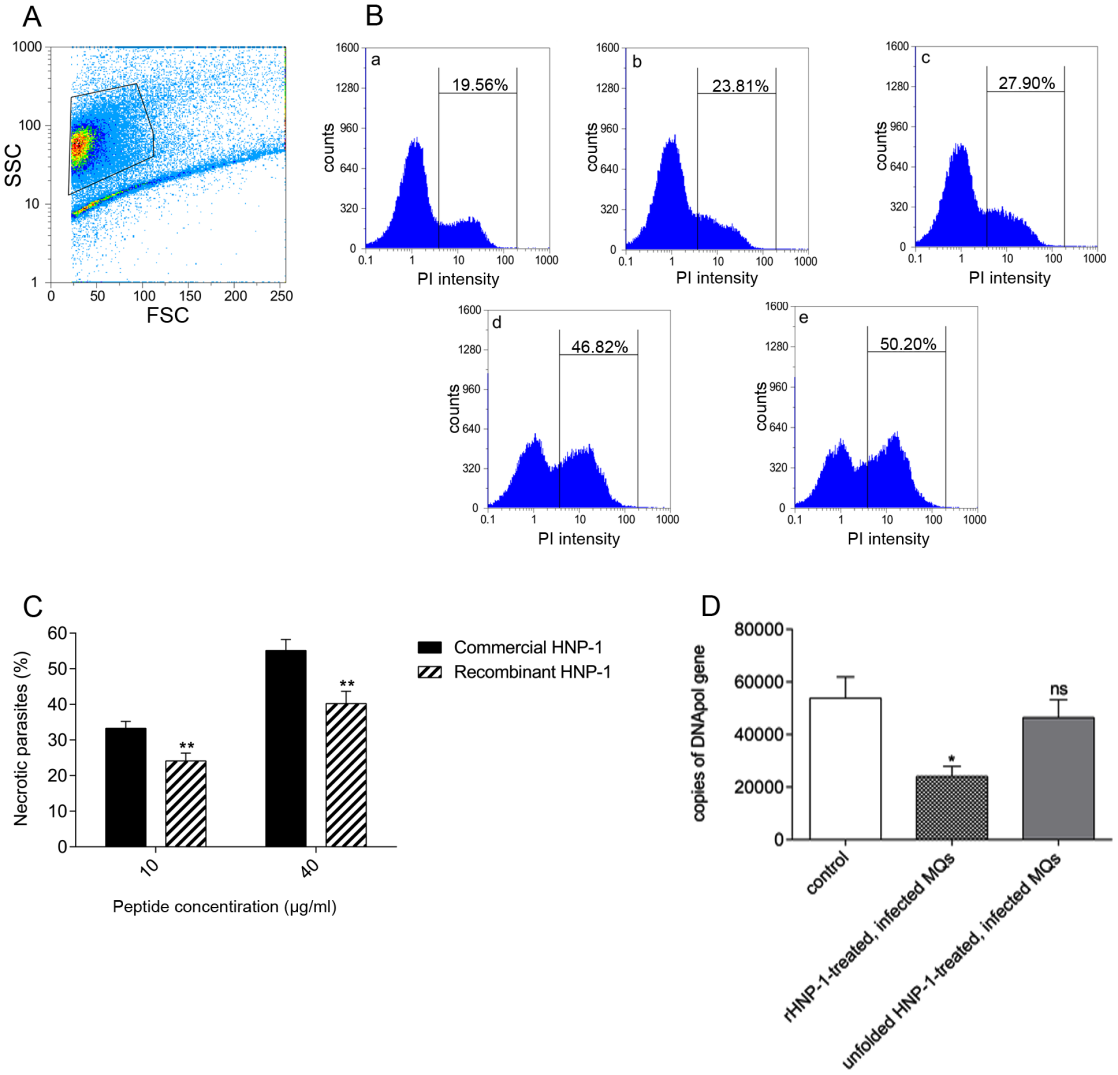
Anti-promastigote and anti-amastigote effects of rHNP-1. A) Initial gating of stationary-phase promastigotes. B, C) Anti-promastigote effect of commercial and recombinant HNP-1. After 24 hours, untreated and treated parasites with different concentrations of rHNP-1 and commercial form were stained with PI followed by flow cytometric analysis. B) By increasing the concentration of rHNP-1 from 0 to 60 µg/ml (a, b, c, d and e related to 0, 10, 20, 40 and 60 µg/ml concentrations of peptide, respectively), the percentage of necrotic population has increased consequently. The figure showed the results of three independent experiments. C) The flow cytometric results of three independent experiments showed that the percentage of necrotic population after being treated with 10 and 40 µg/ml concentrations of rHNP-1 was significantly less than the percentage of necrotic population treated with similar concentrations commercial HNP-1. D) Anti-amastigote effect of rHNP-1. *L. major*-infected LM-1 macrophages were treated by rHNP-1 or unfolded HNP-1. Untreated, infected LM-1 cells were act as control. Extracted total genomic DNA from different groups was subject to real-time PCR assay in order to quantify DNApol and TBP. For each sample, PCR assay was performed in duplicate, and the experiment was performed three times. DNApol quantity was normalized to TBP quantity of each sample. Real-time PCR analysis revealed that the normalized DNApol quantity in rHNP-1-treated LM-1 cells was significantly less than untreated ones (*p*<0.05) and there is no significant difference between DNApol quantities in unfolded HNP-1-treated and untreated groups.

The ability of rHNP-1 to kill stationary-phase promastigotes of *L. major* was evaluated and compared with commercial HNP-1. Stationary-phase promastigotes, incubated with different concentrations of recombinant (ranging from 0 to 60 µg/ml) and commercial HNP-1 (two distinct concentrations; 10 and 40 µg/ml), were stained with PI and subject to FACS analysis in order to determine changes in the PI uptake. As shown in Figure 2B, the higher the concentration of rHNP-1, the higher frequency of necrotic cells. We found that the commercial HNP-1 (at concentrations of 10 and 40 µg/ml) was 1.4-fold more potent than rHNP-1 (as illustrated in Figure 2C, *p*<0.01).

### Anti-amastigote effect of rHNP-1 on infected macrophages

The ability of rHNP-1 to kill *L. major* amastigote was evaluated by quantifying the DNApol gene by real-time PCR in rHNP-1-treated LM-1 cells and compared with parasites treated with unfolded HNP-1 and untreated cells. As shown in Figure 2D, real-time PCR analysis revealed that the normalized DNApol quantity in rHNP-1-treated LM-1 cells was significantly less than untreated ones (24018±3857gene copies versus 53867±8040gene copies, *p*<0.05) confirming anti-amastigote activity of rHNP-1. However there was no significant difference between DNApol quantities in unfolded HNP-1-treated and untreated groups which was obviously representative of a direct correlation between antimicrobial activity and peptide folding.

### rHNP-1 is not cytotoxic for PMNs and murine macrophages

**Figure 3:**
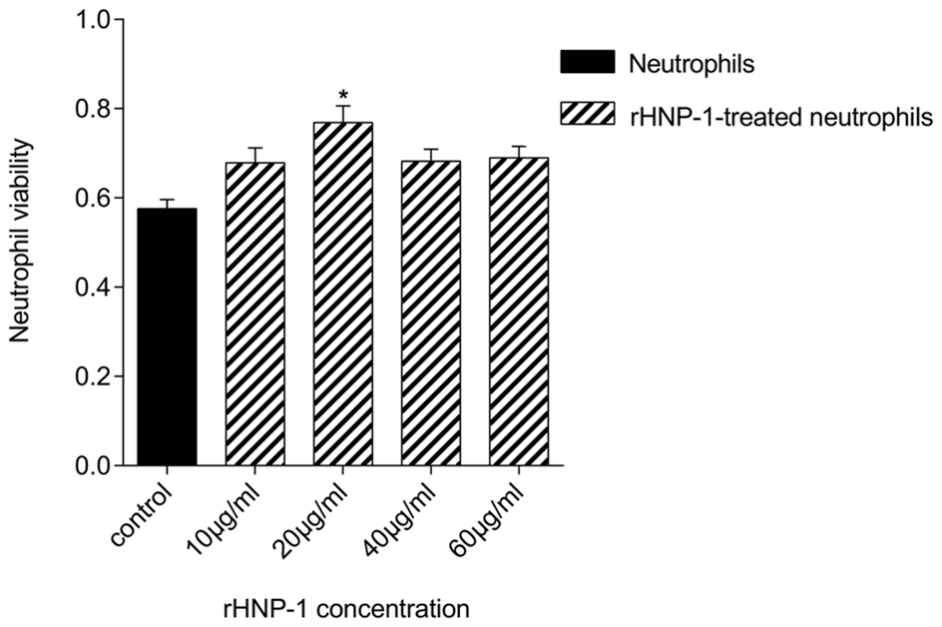
rHNP-1 increased neutrophil viability. MTT assay of treated neutrophils with different concentrations of rHNP-1 (in the range of 0 (as control group) to 60 µg/ml) revealed that rHNP-1 increased neutrophil viability in comparison with control. Concentration of 20 µg/ml had the highest effect on neutrophil viability. Experiment was performed three times in triplicate. Data were represented as mean ± SD. * *p*<0.05 versus control.

The concentrations of rHNP-1 (10, 20, 40 and 60 µg/ml) used, had no cytotoxic effect on neutrophils. On the contrary, they tended to increase the cell viability compared to control. As shown in Figure 3, the highest effect was related to the concentration of 20 µg/ml, so was selected as optimal for further studies. In the case of LM-1 cells, we found that the concentrations above 20 µg/ml of rHNP-1 were toxic, but lower concentrations (10 and 20 µg/ml) did not affect cell viability compared to control (data not shown).

### rHNP-1 influences neutrophil apoptosis

**Figure 4:**
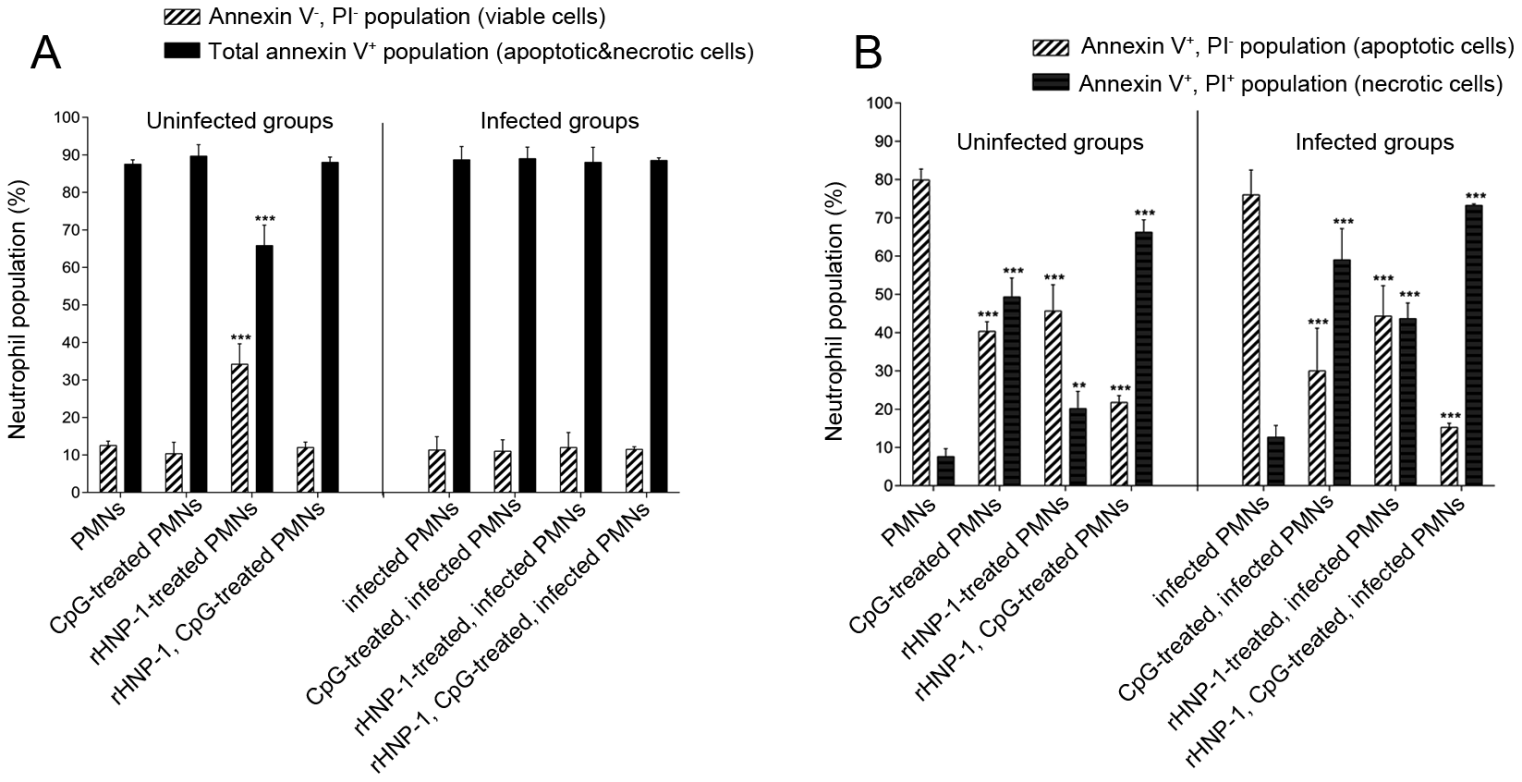
Assessment of neutrophil apoptosis by flow cytometry. Isolated neutrophils from healthy individuals (either *in vitro Leishmania*-infected or not) were treated by 20 µg/ml of rHNP-1 (in presence or absence of 20 µg/ml of class A CpG motif). Apoptosis was assessed by flow cytometry using FITC–annexin V and PI. 15000 events were counted per sample and the percentages of apoptotic, dead or viable cells were determined. This assay was performed on isolated PMNs from 10 healthy individuals (in ten separate experiments) in duplicate. Values are compared between treated (by rHNP-1 and/or CpG motif) and untreated neutrophils and each bar represents the mean ± SD of ten independent experiments. * 0.01<*p*<0.05, ** 0.001<*p*<0.01 and *** *p*<0.001 versus control of each group (uninfected or infected) A) After rHNP-1 treatment of uninfected neutrophils, the percentage of viable cells increased. In all other treated groups, the percentage of viable cells (and consequently total Annexin V^+^ cells) was the same as untreated groups. B) Apoptotic cells and dead cells had different percentages in total Annexin V^+^ population resulted in an apoptotic to dead ratio of more than 6 for uninfected and infected control groups to a ratio of less than 2.5 for all treated groups.

The lifetime of neutrophils is relatively short and they undergo spontaneous apoptosis, which can be inhibited by various pathogen- and host-derived substances. Here, we investigated whether rHNP-1 (as a host derived substance) and/or CpG (as a potent immunostimulator) could influence neutrophil apoptosis. Most neutrophils (85%), either infected or uninfected, were Annexin V^+^ (i.e. apoptotic and dead population) after 18 hours incubation (Figure 4A). Following rHNP-1 treatment of uninfected neutrophils, the percentage of Annexin V^+^ population was significantly reduced compared to untreated, uninfected and infected neutrophils (66±5% versus 88±1% and 89±3%, respectively, *p*<0.001, Figure 4A and Figure S3). This indicates that rHNP-1 increases the lifespan of uninfected neutrophils. Except for rHNP-1 treatment of uninfected neutrophils, the other treatments did not have any effect on the percentage of viable cells (Figure 4A). Furthermore, the percentages of Annexin V^+^, PI^−^ population (apoptotic cells) and Annexin V^+^, PI^+^ population (dead cells) in all treated groups were significantly different from controls (Figure 4B). In untreated neutrophils, 79.9±2.8% and 76±6.5% for uninfected and infected groups, respectively, were Annexin V^+^, PI^−^ (apoptotic). Following different treatments, parallel with decreasing in apoptotic cells, an increase in the frequency of dead cells (Annexin V^+^, PI^+^ population) was seen (irrespective of being infected or not (Figure 4B)). Adding CpG motif or rHNP-1 separately to neutrophils (either infected or not) caused significant decrease in apoptotic population (40±3% and 46±7% for uninfected groups and 30±11% and 44±8% for infected groups, respectively, *p*<0.001) and adding the combination of them caused the maximum decrease in the frequency of apoptotic cells (22±1% and 15±1% for uninfected and infected groups, respectively, *p*<0.001), showing their synergistic effect on neutrophil death. The addition of rHNP-1 increased viability of neutrophils (34±5, *p*<0.001) confirming MTT assay result, while in all other groups, the percentage of viable cells was the same as control groups (≈11.5%).

### rHNP-1 changes *Leishmania*-induced cytokine production in PMNs

To further investigate the activities of rHNP-1 and/or CpG motif on human neutrophils, we evaluated their abilities to induce production of the cytokines TNF-α, IL-8 and TGF-β under different conditions. To increase cell viability and delay apoptosis, neutrophils were first treated by 50 ng/ml of GM-CSF. This procedure will also amplify cytokine production to the detection range of ELISA kits used. GM-CSF-treated neutrophils (either infected or not) were stimulated by rHNP-1 and/or CpG motif for 18 hours. The cytokines of interest were then quantified in cell-free supernatants by ELISA.

**Figure 5:**
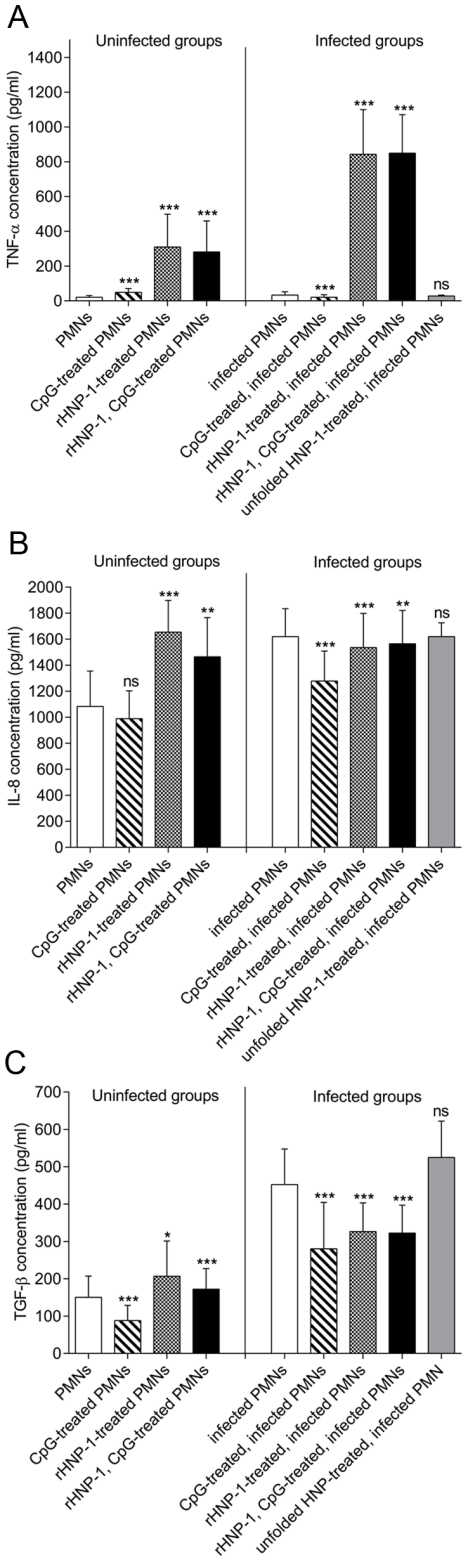
Effect of rHNP-1 and/or CpG motif on cytokine production from uninfected and infected neutrophils. GM-CSF-treated neutrophils (either *in vitro Leishmania*-infected or not) were treated by rHNP-1 (in presence or absence of class A CpG motif). Culture supernatants were then collected and the levels of TNF-α (A), IL-8 (B) and TGF-β (C) were quantified by ELISA. This procedure was carried out separately on isolated neutrophils from 20 healthy individuals and the assay was performed in duplicate. Each bar represents mean ± SD. * 0.01<*p*<0.05, ** 0.001<*p*<0.01 and *** *p*<0.001 versus control of each group (uninfected or infected). ns: not significantly different from control A) Cell treatment by rHNP-1 or combination of rHNP-1 with CpG motif substantially increased TNF-α production from neutrophils (irrespective of being infected). B and C) rHNP-1 or its combination with CpG motif had different effect on IL-8 or TGF-β release from uninfected and infected cells; an increasing effect on uninfected neutrophils and decreasing effect on infected ones. CpG treatment of infected groups had a reduction effect on IL-8 and TGF-β productions.

As shown in Figure 5A, rHNP-1 markedly induced production of TNF-α in uninfected neutrophils, (310±188 pg/ml versus 20±10 pg/ml, *p*<0.001). Furthermore, rHNP-1 exerted its highest effect on infected neutrophils leading to a 3-fold increase in TNF-α concentration as compared with its effect on uninfected neutrophils (843±257 pg/ml versus 310±188 pg/ml, *p*<0.001). Although, treatment of uninfected and infected cells by CpG motif alone increased and decreased TNF-α production respectively (*p*<0.001), the value of this cytokine in the cultures of these groups was less than 50 pg/ml. Co-administration of rHNP-1 with CpG motif did not cause any significant change in TNF-α concentration compared to neutrophils treated by rHNP-1 alone (*p*>0.05).

rHNP-1 treatment of uninfected neutrophils (regardless of CpG motif treatment) primed the release of IL-8 and reduced IL-8 production from infected neutrophil culture (Figure 5B, *p*≤0.01). CpG motif alone had a decreasing effect on IL-8 release from infected neutrophils (1279±230 pg/ml versus 1621±214 pg/ml, *p*<0.001) and had no significant effect on uninfected neutrophils. The reduction effect of CpG motif on IL-8 production from infected neutrophils was significantly higher than rHNP-1 (*p*<0.001).

The highest concentration of TGF-β was detected in untreated, parasite-infected neutrophils (Figure 5C), equal to 452±95 pg/ml (*p*<0.001 versus uninfected neutrophils). Whereas rHNP-1 enhanced TGF-β release from uninfected neutrophils (207±94 pg/ml versus untreated neutrophils; 151±57 pg/ml, *p*≈0.04), it attenuated the TGF-β release from infected ones (326±77 pg/ml versus 452±95 pg/ml, *p*<0.001). Further stimulation with CpG motif and rHNP-1 had similar effect as rHNP-1 alone; increasing TGF-β release in uninfected group (172±55 pg/ml versus 151±57 pg/ml, *p*<0.001), while decreasing a release from infected group (322±75 pg/ml versus 452±95 pg/ml, *p*<0.001). CpG treatment reduced TGF-β up to 60% in both uninfected and infected groups (compared to uninfected or infected control, *p*<0.001).

To summarize, rHNP-1 (or its combination with CpG motif) markedly induced production of TNF-α, while an inhibitory effect was seen on both IL-8 and TGF-β productions in *Leishmania*-stimulated cultures. Treatment of infected neutrophils by unfolded HNP-1 was acting as negative control and concentrations of different cytokines in this culture did not differ from those in untreated, infected neutrophil culture.

### rHNP-1 treatment reduces *Leishmania* infectivity of PMNs

To evaluate if rHNP-1 had effect on the *Leishmania* infectivity rate of PMNs, the DNApol gene of rHNP-1-treated, *Leishmania*-infected neutrophils was quantified by real-time PCR and compared to untreated group.

**Figure 6:**
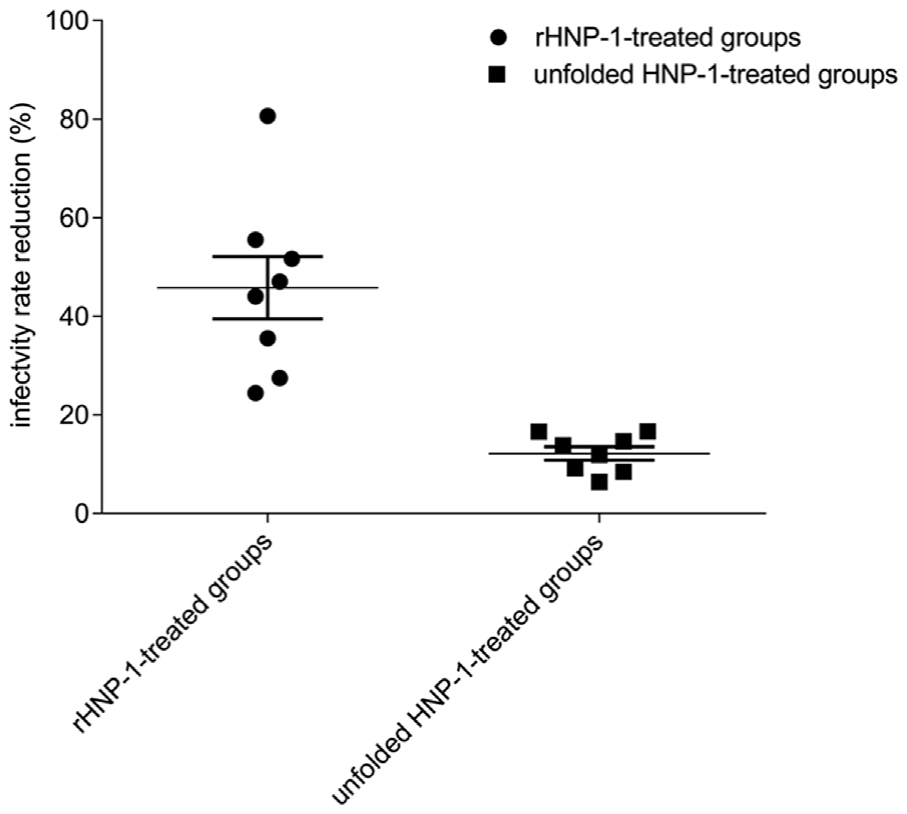
rHNP-1 treatment of infected neutrophils caused a significant reduction in parasite infectivity, determined by real-time PCR. *L. major*-infected neutrophils were treated by rHNP-1 or unfolded HNP-1. Untreated, infected neutrophils were as control. Extracted total genomic DNA from different groups was subject to real-time PCR assay in order to quantify DNApol. As a negative control, reactions without DNA template were also performed. For each sample, PCR was performed in duplicate, and the experiment was performed three times. The percentage of infectivity rate reduction was determined based on following equation: [1−(DNApol quantity_treated group_/DNApol quantity_control group_)]×100. rHNP-1 caused infectivity rate reduction in 8 treated neutrophil samples (out of 10) in the range of 20% to 80% and a mean ± SD value of 45.79±17.90%. Significant difference was found in the mean percentages of infectivity rate reduction between rHNP-1-treated neutrophil samples and unfolded HNP-1-treated ones (46±18% versus 12±4%, *p*≈0.001), showing that antimicrobial activity of HNP-1 is relied on its correct folding.

We found that infectivity rate was reduced in 8 out of 10 rHNP-1-treated neutrophil samples, with a decrease of 20 to 80% (Figure 6). Infectivity rate reduction between rHNP-1-treated and unfolded HNP-1-treated groups was significantly different (46±18% versus 12±4%, *p*≈0.001), showing a correct folding dependence of antimicrobial activity of HNP-1.

## Discussion

All currently used first-line and second-line drugs for the treatment of leishmaniasis have drawbacks in terms of toxicity, cost and resistance. Because of low susceptibility to resistance and being nontoxic to host cells at effective concentrations against pathogens, AMPs are attractive candidates. Despite the extensive documentation on the activities of AMPs against bacteria and fungi, studies investigating the effects of AMPs against protozoa are rare and to the best of our knowledge there is no report on HNP-1 tested against *L. major*.

Pharmacological studies of defensins needs relatively large quantities of pure functional peptides. Since the purification process of natural peptides is difficult and chemical synthesis of these peptides is expensive, recombinant strategies are a promising alternative. By using bacterial expression system, we succeeded in obtaining substantial amounts of peptide, which was purified on Ni-NTA column via FPLC. In contrast to the commercial form of HNP-1, the purified peptide needed folding in order to become bioactive. By using the method of Rehder and Borges for *in vitro* folding of proteins with disulfide bonds [26], we succeeded in producing active recombinant peptide with a functional anti-bacterial activity, albeit lower than that of commercial HNP-1.

We found that our rHNP-1 was active against stationary-phase promastigotes of *L. major*. By flow cytometric analysis, we observed that increased levels of rHNP-1 caused necrosis of parasites, which reached to 50% at a concentration of 60 µg/ml (Figure 2B). While the commercial HNP-1 was slightly more potent in parasite killing (1.4-fold higher), the microbicidal activity of in house recombinant HNP-1 was deemed to be sufficient and due to cost-effectiveness preferred for further studies. The concentration needed to kill 50% of parasites (about 60 µg/ml) is higher than that needed to kill 50% of bacteria (less than 7 µg/ml). This may be due to the complicated membrane structure of the parasite; extremely rich in glycosylphosphatidylinositol-anchored molecules such as leishmanolysin (the major surface-metalloprotease) and LPG, which are considered as protective agents for parasites.

Since the activities of AMPs may vary against amastigotes; the pathological form of *Leishmania* in vertebrate host [28], the anti-amastigote activity is important to be determined for further drug development. To determine the effect of rHNP-1 on *L. major* amastigotes, we measured the amastigote laden in rHNP-1-treated and untreated LM-1 macrophages. The quantification was by using DNApol as the target gene of parasite. Prina *et al.* have shown that *Leishmania* DNA degradation follows rapidly after parasite death [29]. Thus the quantified DNA was related to the number of viable amastigotes. Based on our results and previous documents that denatured HNP-1 does not display antimicrobial activity [30], as a control, we tested the effect of unfolded HNP-1 on the parasite load in LM-1 macrophages. While rHNP-1 treatment significantly reduced the amastigote load (24018±3857 gene copies versus 53867±8040 gene copies, *p*<0.05), unfolded HNP-1 had no significant effect, similar to control (Figure 2D), indicating that anti-parasitic effect of rHNP-1 (like its anti-bacterial effect) relies on a correct tertiary structure. The mechanism by which rHNP-1 reduces amastigote load in macrophages is unknown, but may be a direct effect of rHNP-1 on parasites. In this respect, Arnett *et al.* have shown that HNP-1 is taken up by bacteria-infected macrophages and reduces bacterial load [31].

Neutrophils play an important role as host cells in the early phase of *L. major* infection. Therefore, agents that modulate neutrophil functions may alter the outcome of infection and/or vaccination. In addition to its potent antimicrobial activity, HNP-1 has immunomodulatory effects; stimulating various immune cells to production of cytokines. HNP-1 has been shown to act as a chemotactic factor for both macrophages and T lymphocytes [11] and HNP-1 treatment enhances leukocyte accumulation at the site of infection in *K. pneumonia*-infected mice [30] and provokes TNF-α release from lymphocytes [32]. Further, HNP-1 treatment of macrophages has been shown to inhibit proliferation of the intracellular bacteria [31]. Another immunomodulatory molecule is CpG motif. It has been shown that CpG motif administration to mice causes neutrophil accumulation at the site of infection [33], [34] and induction of IL-8 secretion [35]. In our previous study, we showed considerable level of TNF-α production by class A CpG-treated neutrophils following GM-CSF treatment [24]. Further, class A CpG motif is considered as a potent stimulator of Th1 response [36].

To follow up on the anti-parasitic effect of rHNP-1, we tested the immunomodulatory effect of rHNP-1 on parasite-infected neutrophils combined with class A CpG motif co-stimulation. Treatment of neutrophils with rHNP-1 was found to delayed the apoptosis of uninfected neutrophils, which is in concordance with the study by Nagaoka *et al.* [37]. However, rHNP-1 treatment had no effect on viability when neutrophils had been infected with *L. major*. Nagaoka *et al.* showed that the anti-apoptotic effect of rHNP-1 may be related to its effect on the expression of truncated Bid and Bcl-x_L_ (considering as pro-apoptotic and anti-apoptotic proteins) and consequently caspase 3 activity (a key executor of apoptosis) [37]. Why rHNP-1 had no anti-apoptotic effect on infected neutrophils is not clear, but it could be possible that parasite antagonizes the signaling pathway through which rHNP-1 exerts its anti-apoptotic effect. We suggest that rHNP-1, rather than blocked, delayed the neutrophil apoptosis for up to 24 hours. In support of this, we observed that the number of viable treated cells dropped during the second day of culture (data not shown). Contrary to reports suggesting that CpG motif delays neutrophil apoptosis [35], [38], we found no such effect of CpG motif alone or in combination with rHNP-1 (Figure 4A). This controversy over CpG results may be due to the different kinds of CpG motif used in our experiments and mentioned studies; they used *E. coli* DNA for stimulating neutrophils, while we used class A CpG motif. It was reported that short oligonucleotides are generally less potent immunostimulators than *E. coli* DNA [39], [40].

A noteworthy similarity among all treated neutrophils is reduction in the ratio of apoptotic to dead cells in comparison with control (Figure 4B). Two possible mechanisms could be considered for this reduction in the ratio of apoptotic to dead cells. First, rHNP-1 and/or CpG treatment stimulated the signaling pathways required for transition from early apoptosis to secondary necrosis and consequently accelerated this step (as compared with untreated groups). Second possible mechanism is that such treatments partly blocked apoptosis pathway and drove cell death by necrosis rather than apoptosis. It seems that these dead cells are usually better at triggering inflammatory responses and altering the lifespan of neutrophils may influence the fate of intracellular *Leishmania* parasite. While necrotic and apoptotic neutrophils are engulfed by macrophages to similar extents [41], neutrophils that die by necrosis, are usually better at triggering inflammatory responses. It has been shown that phagocytosis of apoptotic neutrophils by macrophages, contrary to necrotic bodies, fails to trigger antimicrobial effector functions [22], [42], [43]. The implication of these finding on our results are not clear, as necrosis as well as prolonged neutrophil survival, which maybe represent enhanced neutrophil function, could be envisaged to have beneficial effects for the host.

We therefore sought to investigate how rHNP-1 and CpG motif affected cytokine/chemokine production by neutrophils. For this purpose, we measured TNF-α, IL-8 and TGF-β concentrations in culture supernatants. rHNP-1 had significantly higher effect than CpG motif on induction of TNF-α production by neutrophils and there was no additive effect of CpG motif (Figure 5A). An additive effect on TNF-α production was seen when rHNP-1 was added to *L. major*-infected neutrophils; parasite itself caused a 1.6-fold increase in TNF-α production over background, while rHNP-1 treatment of infected neutrophils exhibited a 25.6-fold increase (*p*<0.001). TNF-α is a pluripotent cytokine, which in the context of *Leishmania* infection is primarily linked to protection. Thus, enhanced TNF-α would be expected to be beneficial to the host.

IL-8 is a chemokine important in recruitment of inflammatory cells. We found, in concordance with Laufs *et al.* and Zandbergen *et al.* [44], [45], a small (1.5-fold) but significant (*p*<0.001) increase of IL-8 in parasite-infected neutrophil culture in comparison with uninfected control. While both rHNP-1 and CpG motif showed a reduction effect on IL-8 production in parasite-infected neutrophils, the latter had more potent effect. Similar to the effect on IL-8 production, we found that parasite uptake by neutrophils stimulated TGF-β production, an immunosuppressive cytokine associated with susceptibility to *Leishmania* infection [46]. Interestingly, rHNP-1 and CpG treatment of infected neutrophils reduced the level of TGF-β up to 28% and 38% respectively (*p*<0.001).

The reduced *Leishmania* infectivity rate, observed following rHNP-1 treatment of neutrophils, maybe correlated with the marked effect of rHNP-1 on TNF-α production, a pro-inflammatory cytokine potentiating neutrophil microbicidal capacity. Moreover, This reduction may be explained by direct leishmanicidal effect of rHNP-1 on entrapped promastigotes in neutrophils, since *Leishmania* parasites do not fully transform into amastigotes inside neutrophils [22], [42]. However, for this direct effect, HNP-1 must be transported into the neutrophils and trafficked to the parasitophorous vacuole (containing parasites) or interact with the releasing parasites from the parasitophorous vacuole to carry out direct leishmanicidal activity. Alternatively, it is possible that HNP-1, as may be suggested by the induction of TNF-α production, triggers mechanisms in neutrophils and thereby indirectly potentiates their ability to kill parasites.

In conclusion, HNP-1 and similar peptides may represent a promising alternative in the search for new nontoxic, broad-spectrum antibiotic agents. Due to defensins' potency to promote development of protective immune responses, they can be regarded as adjuvants. By optimizing simple methods for producing active form of AMPs including HNP-1, new horizons for further investigation on functional, structural and pharmacological features are open. Better understanding of the properties and mechanisms underlying immunomodulatory effects of rHNP-1 on neutrophils may benefit the design of new and improved leishmanicidal derivatives with high selectivity for the pathogens and/or with potent immune-adjuvant effects. Engagement of neutrophils may enhance the killing of *Leishmania*, and facilitate initiation of a proper immune response against *Leishmania* infection.

## Supporting Information

Figure S1 **Isolation of total RNA from PMNs of healthy individuals and PCR amplification of HNP-1 gene.** A) Assessment of isolated total RNA by gel electrophoresis on a 1% agarose gel. 1 & 2) RNA from healthy volunteers' neutrophils. B) cDNA obtained from isolated RNA was amplified by PCR assembly. The PCR product was evaluated on a 2% agarose gel electrophoresis which exhibited an expected single band of 123 bp. Left to right: Molecular weight marker, HNP-1 gene.
(TIF)

Figure S2 **Profiles of the anti-** ***E. coli*** **activity vs. concentration for the commercial and recombinant HNP-1.** The anti-bacterial activity of rHNP-1 and commercial HNP-1 were determined using a standard assay against *E. coli* (ATCC 25922) described by Pazgier and Lubkowski [27]. As the bacteria inhibition curve illustrates in this figure, the growth of bacteria was dramatically suppressed with the increasing concentrations of rHNP-1 and its commercial form, which demonstrated that both of them were bioactive. The data represent mean ± SD for three independent experiments.
(TIF)

Figure S3 **rHNP-1 delayed neutrophil apoptosis.** Isolated, uninfected neutrophils were treated by 20 µg/ml of rHNP-1. Apoptosis was assessed by flow cytometry using FITC–annexin V and PI. 15000 events were counted per sample and the percentages of apoptotic, dead or viable cells were determined. This assay was performed on isolated PMNs from 10 healthy individuals (in ten separate experiments) in duplicate. After rHNP-1 treatment of uninfected neutrophils, the percentage of viable cells increased in comparison with control (39.87% versus 11.30%).
(TIF)
