# Supplementary figures and images for: Human Neutrophil Peptide-1 (HNP-1): A New Anti-Leishmanial Drug Candidate
Source: PLoS Negl Trop Dis. 2013 Oct 17;7(10):e2491. doi: 10.1371/journal.pntd.0002491 (PMC3798388; doi:10.1371/journal.pntd.0002491)

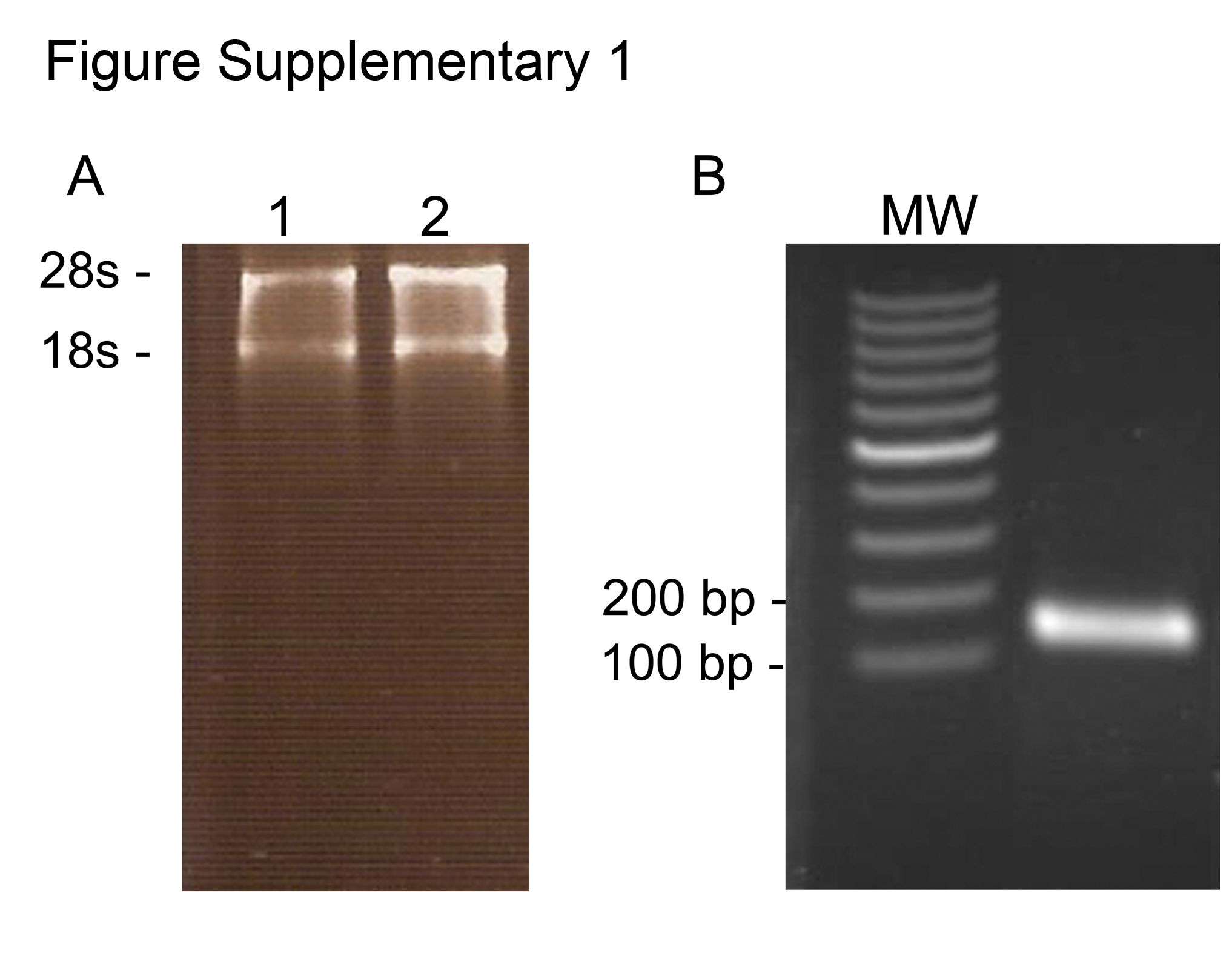

Supplement: Figure S1 — Isolation of total RNA from PMNs of healthy individuals and PCR amplification of HNP-1 gene. A) Assessment of isolated total RNA by gel electrophoresis on a 1% agarose gel. 1 & 2) RNA from healthy volunteers' neutrophils. B) cDNA obtained from isolated RNA was amplified by PCR assembly. The PCR product was evaluated on a 2% agarose gel electrophoresis which exhibited an expected single band of 123 bp. Left to right: Molecular weight marker, HNP-1 gene. (TIF) [file pntd.0002491.s001.tif]

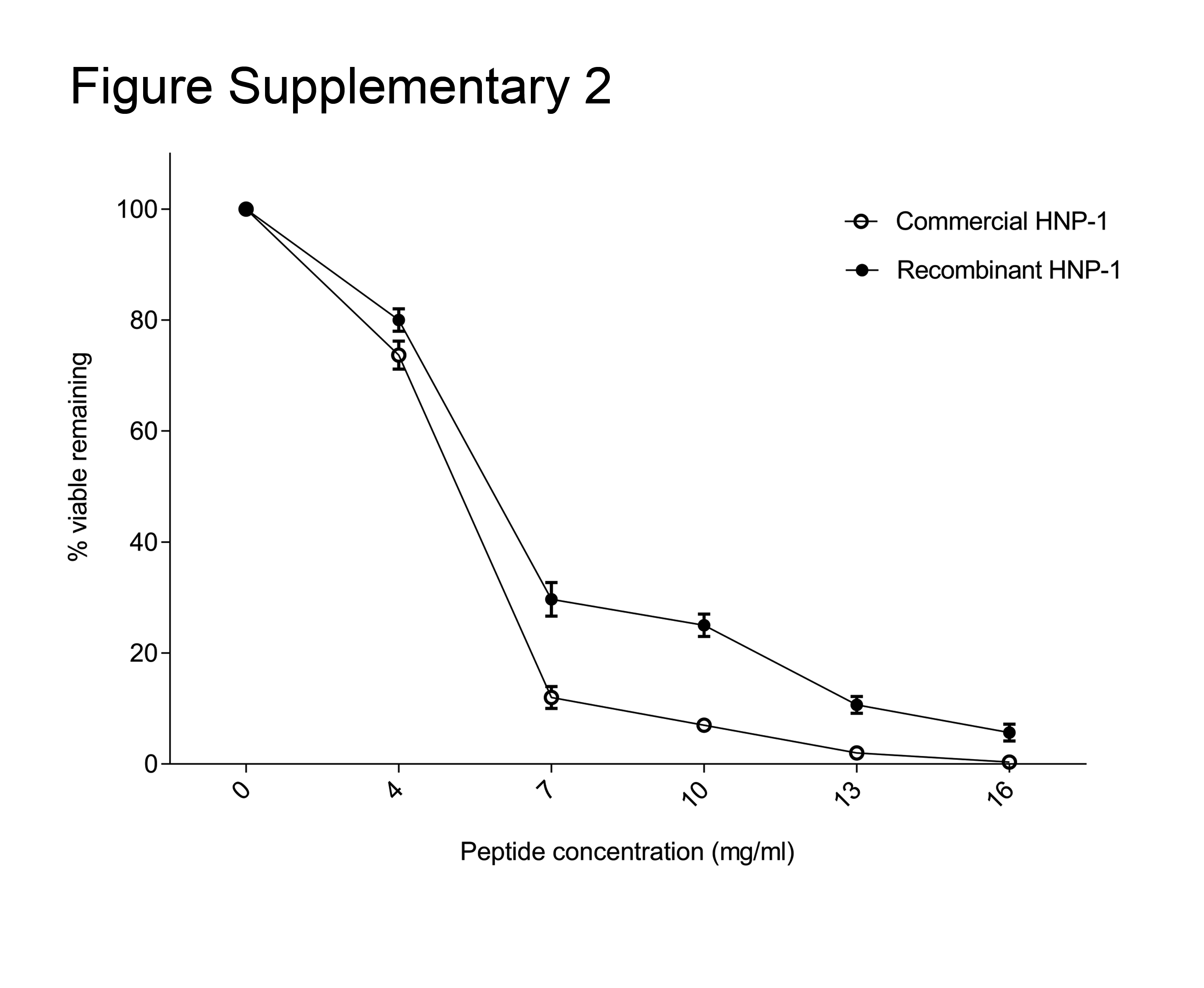

Supplement: Figure S2 — Profiles of the anti- E. coli activity vs. concentration for the commercial and recombinant HNP-1. The anti-bacterial activity of rHNP-1 and commercial HNP-1 were determined using a standard assay against E. coli (ATCC 25922) described by Pazgier and Lubkowski [27]. As the bacteria inhibition curve illustrates in this figure, the growth of bacteria was dramatically suppressed with the increasing concentrations of rHNP-1 and its commercial form, which demonstrated that both of them were bioactive. The data represent mean ± SD for three independent experiments. (TIF) [file pntd.0002491.s002.tif]

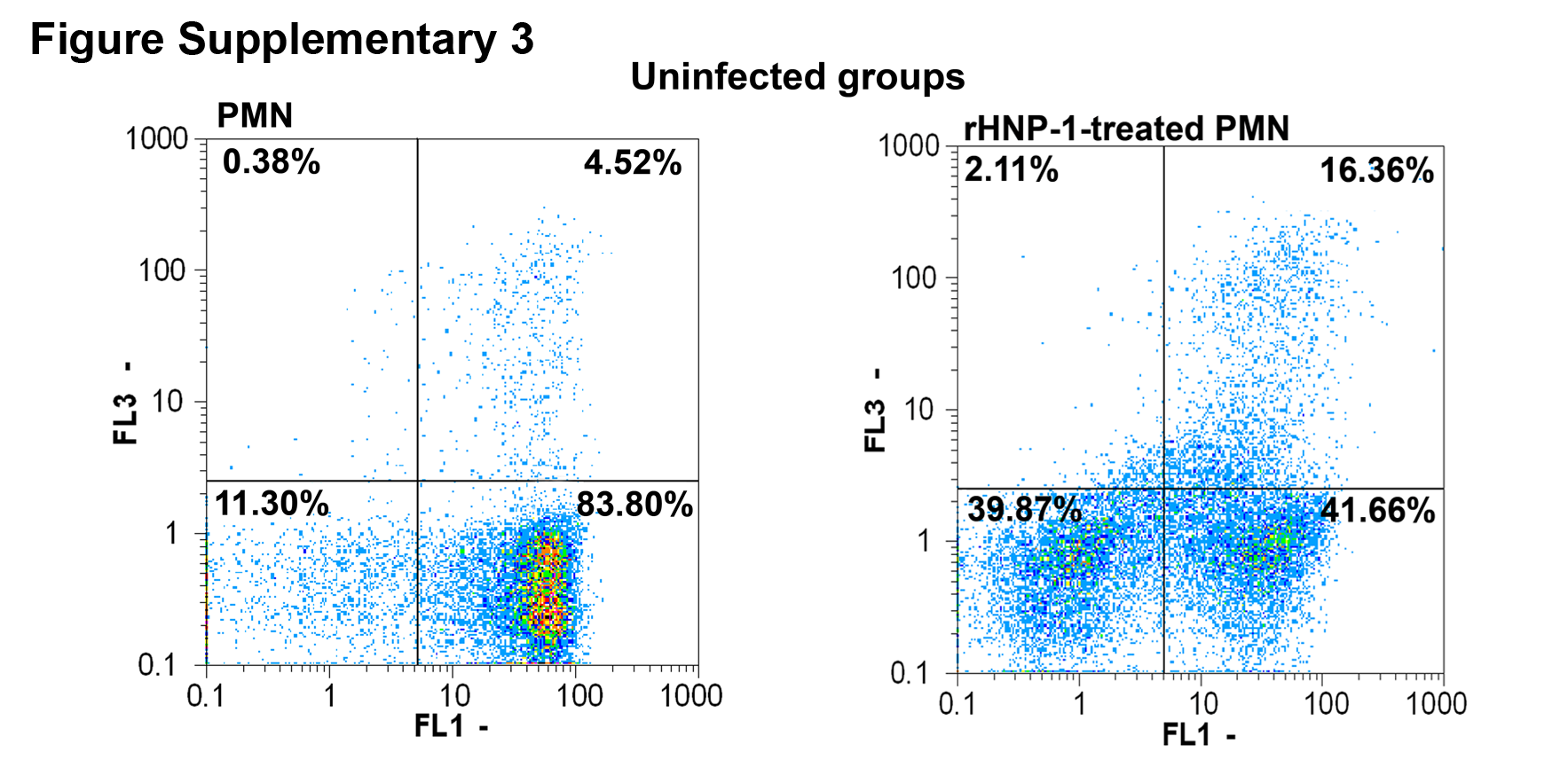

Supplement: Figure S3 — rHNP-1 delayed neutrophil apoptosis. Isolated, uninfected neutrophils were treated by 20 µg/ml of rHNP-1. Apoptosis was assessed by flow cytometry using FITC–annexin V and PI. 15000 events were counted per sample and the percentages of apoptotic, dead or viable cells were determined. This assay was performed on isolated PMNs from 10 healthy individuals (in ten separate experiments) in duplicate. After rHNP-1 treatment of uninfected neutrophils, the percentage of viable cells increased in comparison with control (39.87% versus 11.30%). (TIF) [file pntd.0002491.s003.tif]
